# Herpes labialis and Nigerian dental health care providers: knowledge, attitudes, behaviors, and refusal to treat

**DOI:** 10.1186/s12913-015-1023-9

**Published:** 2015-09-15

**Authors:** Clement Chinedu Azodo, Agnes O. Umoh

**Affiliations:** Department of Periodontics, School of Dentistry, University of Benin, Ugbowo, Benin City, Edo State 300001 Nigeria

## Abstract

**Background:**

The few existing studies on herpes labialis among health care workers have been predominantly among non-dental health care workers. The purpose of this study was to determine Nigerian dental health care providers’ knowledge of, attitudes toward, preventive behaviors for, and refusal to treat patients with herpes labialis.

**Methods:**

This cross-sectional study was conducted among final-year dental students at the University of Benin, dental house officers, and residents at the University of Benin Teaching Hospital, Benin City, Nigeria. Data collection was *via* a self-administered questionnaire. Bivariate statistics and logistic regression were used to relate the dependent and independent variables.

**Results:**

Of the 120 questionnaires distributed, 110 were completed and returned, giving a 91.7 % retrieval rate. However, 15 of the returned questionnaires were discarded because they were improperly completed, leaving a total of 95 questionnaires for final analysis in this study. The majority of participants were over 28 years old (54.7 %), male (67.4 %), unmarried (66.3 %), and postgraduate dental health care providers (51.6 %). Less than half (43.2 %) of participants demonstrated adequate overall knowledge of herpes labialis. About one-tenth (10.5 %) and more than three-quarters (87.4 %) of participants reported a positive attitude and performance of adequate preventive behaviors, respectively. A total of 16.8 % of participants reported a high tendency to refuse treatment to patients with herpes labialis. Although not statistically significant, young, unmarried, male undergraduate participants reported a greater likelihood to refuse treatment to herpes labialis patients. We found a statistically significant positive correlation between attitude and refusal to treat patients with herpes labialis. However, marital status and the attitude of participants toward these patients emerged as the determinants for refusal to treat patients with herpes labialis.

**Conclusions:**

Data from this study revealed a high level of inadequate knowledge, negative attitudes, and reasonably adequate preventive behaviors with respect to herpes labialis. One out of every six dental health care workers studied reported having refused to treat patients with herpes labialis. Unmarried dental health care providers and those with negative attitudes toward herpes labialis patients were more prone to refuse treatment to these patients.

**Electronic supplementary material:**

The online version of this article (doi:10.1186/s12913-015-1023-9) contains supplementary material, which is available to authorized users.

## Background

Herpes labialis is a recurrent, self-limiting, infectious, vesiculo-ulcerative herpes simplex virus (HSV) infection that usually affects the lips and adjacent skin. The condition is considered a global problem [1]. It is usually transmitted by direct contact with the lesions or body fluids of an infected individual, although transmission through skin-to-skin contact during periods of asymptomatic HSV shedding is also possible. These modes of transmission pose a serious risk to dental health care professionals for infection with herpes labialis. The literature includes reports of herpes whitlow and herpes keratitis in dental health care providers after treating patients with active herpes lesions, in the absence of proper infection control practices [2, 3]. Although barrier protection methods are the most reliable manner of preventing herpes infection, these do not completely eliminate the risk of transmission. It is therefore anticipated that the risk of infection influences the willingness of dental health care workers to treat patients with herpes labialis, in the same way as when treating patients with other bloodborne viral infections.

Previous reports of unwillingness by dentists to treat patients with infectious diseases have mainly been related to infection with human immunodeficiency virus (HIV), hepatitis B virus (HBV) and hepatitis C virus (HCV) [4–7]. Refusal to treat patients with HIV has primarily been associated with a lack of ethical responsibility and fears related to cross infection [4]. It has been documented that dental students with poorer knowledge significantly preferred not to treat intravenous drug users and patients with hepatitis because of a perceived higher risk of infection [8].

Prominent herpes labialis lesions on the lip and associated pain alter physical appearance, cause psychological problems, interfere with social activities, and consequently impair quality of life [9, 10]. Depression, isolation, fear of rejection, and self-destructive feelings are among the social and psychological consequences of herpes labialis, although these usually lessen over time. The refusal to treat patients with herpes labialis will therefore not only exert a negative impact on the overall health of affected individuals but will also limit dentists’ contribution to infectious diseases control [6]. Such refusal to treat is unethical and may be viewed as discriminatory by the public, thus sowing distrust in the dentist-patient relationship and hampering communication, which will ultimately undermine optimal dental health care delivery [11, 12].

Studies on herpes labialis have been predominantly among non-health care workers, and the few studies in health care workers have involved non-dental health care workers. The only relevant previous study compared knowledge, attitudes and professional behaviors in relation to herpes labialis among dental and dental hygiene students at different academic levels [13]. However, that study assessed neither the determinants for refusal to treat patients with herpes labialis nor the trigger factors among participants. In that study, the null hypothesis was stated thus: no significant difference exists in the refusal to treat herpes labialis patients between participants with adequate and inadequate overall knowledge. Our proposed hypothesis is as follows: a significant difference exists in the refusal to treat herpes labialis patients between participants with adequate and inadequate overall knowledge. The purpose of this study was to determine Nigerian dental health care providers’ knowledge of, attitudes toward, preventive behaviors for, and refusal to treat patients with herpes labialis.

## Methods

### Ethical considerations

The protocol for this study was reviewed and approved by the Ethics and Research Committee of the University of Benin Teaching Hospital in Benin City, Nigeria. Verbal informed consent was obtained from the participants. Participation in the study was voluntary and no incentive was offered.

### Study design

This was a cross-sectional study conducted in June 2014 among final-year dental students of the University of Benin, and dental house officers and residents at the University of Benin Teaching Hospital, Nigeria.

### Sample size

The sample size of 106 was determined using the Cochran formula for sample size calculation in epidemiological studies, based on the unpublished 7.4 % self-reported annual prevalence of herpes labialis in this group [14]. However, the 120 distributed questionnaires eventually compensated for non-response.

### Selection criteria

All final-year dental students at the University of Benin, as well as dental house officers and residents at the University of Benin Teaching Hospital who were available during the study and who consented, were selected. Those who were absent or did not consent to the study were excluded.

### Data collection

The data collection tool used was a 40-item self-administered validated questionnaire (see Additional file 1). The questionnaire was anonymous without any identifiers. A total of 19 items on our questionnaire were obtained from that of Kanjirath et al. [13] which addressed knowledge, attitudes and professional behavior with respect to herpes virus.

Questionnaires were administered to dental students, and dental house officers and postgraduate residents during regular scheduled classes and postgraduate seminars, respectively. Completed questionnaires were collected immediately afterward.

### Data analysis

The data obtained were subjected to descriptive, correlation and binary regression statistical analysis using Statistical Package for the Social Sciences (SPSS) Version 17.0 (SPSS Inc., Chicago, IL, USA). Participant age was categorized based on the previous experience of the authors. Data regarding knowledge, attitude, professional behaviors, and refusal tendency were dichotomized based on mean values obtained from the pilot study. The mean values were rounded up to the nearest whole number and considered to be the lower limits of adequate, positive, and high categories of knowledge/behavior, attitude and refusal to treat, respectively. In the binary regression analysis, refusal to treat was the dependent variable whereas demographic characteristics, overall knowledge, attitude, and professional behavior were independent variables. Statistical significance was set at *p*  < 0.05.

## Results

Of the 120 questionnaires distributed, 110 were returned completed, giving a 91.7 % retrieval rate. However, 15 of the returned questionnaires were discarded because they were improperly filled out, leaving a total of 95 questionnaires for final analysis in this study (Additional file 2).

### Demographic characteristics of participants

The majority of participants were over 28 years old (54.7 %), male (67.4 %), unmarried (66.3 %), and postgraduate dental health care providers (51.6 %) (Table 1).

**Table 1:** Demographic characteristics of participants

Characteristics	Frequency	Percent
	(*n*)	(%)
Age (years)		
≤28	43	45.3
>28	52	54.7
Sex		
Male	64	67.4
Female	31	32.6
Marital status		
Unmarried	63	66.3
Married	32	33.7
Professional status		
Undergraduate	46	48.4
Postgraduate	49	51.6
Total	95	100.0

### Participant knowledge, attitudes, professional behaviors, and refusal to treat

General knowledge about herpes was adequate in 33.7 % of participants and was significantly associated with age, marital status, and professional status. Trigger knowledge was adequate in 56.8 % of participants and was significantly associated with age, sex, and professional status. Knowledge about transmission prevention was adequate in 58.9 % of participants and significantly associated with sex and professional status. Overall knowledge was adequate in 43.2 % of participants and significantly associated with age, sex, marital status, and professional status. A positive attitude was found in 10.5 % of participants and this was not significantly associated with demographic characteristics. Professional behavior was adequate in 87.4 % of participants. The refusal to treat patients with herpes labialis was reported in 16.8 % of participants (Table 2).

**Table 2:** Knowledge of, attitudes toward, professional behaviors for, and refusal to treat patients with herpes labialis among participants

	Age (years)		Sex		Marital status		Professional status		Total
	≤28	>28	Male	Female	Single	Married	U	P	
General knowledge									
Inadequate	83.7	51.9	62.5	74.2	77.8	43.8	91.3	42.9	66.3
Adequate	16.3	48.1	37.5	25.8	22.2	56.2	8.7	57.1	33.7
* p*-value	0.001		0.258		0.001		0.000		
Trigger factor knowledge									
Inadequate	55.8	32.7	34.4	61.3	47.6	34.4	58.7	28.6	43.2
Adequate	44.2	67.3	65.6	38.7	52.4	65.6	41.3	71.4	56.8
* P*-value	0.024		0.013		0.218		0.003		
Transmission prevention knowledge									
Inadequate	51.2	32.7	31.3	61.3	44.4	34.4	54.3	28.6	41.1
Adequate	44.2	67.3	65.6	38.7	52.4	65.6	41.3	71.4	56.8
* P*-value	0.024		0.013		0.218		0.003		
Transmission prevention knowledge									
Inadequate	51.2	32.7	31.3	61.3	44.4	34.4	54.3	28.6	41.1
Adequate	48.8	67.3	68.8	38.7	55.6	65.6	45.7	71.4	58.9
* p*-value	0.069		0.005		0.346		0.011		
Overall knowledge									
Inadequate	79.1	38.5	46.9	77.4	65.1	40.6	78.3	21.7	56.8
Adequate	20.9	61.5	53.1	22.6	34.9	59.4	36.7	63.3	43.2
* p*-value	0.000		0.005		0.023		0.000		
Attitude									
Positive	7.0	13.5	12.5	6.5	9.5	12.5	10.9	10.2	10.5
Negative	93.0	86.5	87.5	93.5	90.5	87.5	89.1	89.8	89.5
* p*-value	0.789		0.368		0.655		0.916		
Behavior									
Inadequate	11.6	13.5	14.1	9.7	14.3	9.4	19.6	6.1	12.6
Adequate	88.4	86.5	85.9	90.3	85.7	90.6	80.4	93.9	87.4
* p*-value	0.305		0.546		0.496		0.049		
Tendency to treat									
Refusal	18.6	15.4	17.2	16.1	22.2	6.3	21.7	12.2	16.8
No refusal	81.4	84.6	82.8	83.9	77.8	93.8	78.3	87.8	83.2
* p*-value	0.676		0.876		0.049		0.217		

*U* undergraduate, *P* postgraduate

### Bivariate correlation of participant demographic characteristics, knowledge, attitudes, behaviors, and refusal to treat herpes labialis patients

Overall knowledge had a significant positive correlation with age, sex, marital status, professional status, general knowledge, trigger knowledge, and prevention knowledge. Although overall knowledge was negatively correlated with attitude and refusal to treat, this was not statistically significant. Refusal to treat only had a significant positive correlation with attitude (Table 3).

**Table 3:** Bivariate correlation of demographic characteristics, knowledge, attitudes, behaviors, and refusal to treat patients with herpes labialis among participants

Variable		Age	Sex	Marital status	Professional status	Overall knowledge	General knowledge	Trigger knowledge	Prevention knowledge	Attitude	Behavior	Refusal to treat
Age	r	1	-.179	.469^a^	.473^a^	.408^a^	.335^a^	.232^b^	.187	.105	-.027	-.043
	*p*-value		.083	.000	.000	.000	.001	.023	.070	.310	.792	.680
Sex	r	-.179	1	.122	-.089	-.289^a^	-.116	-.255^b^	-.286^a^	-.092	.062	-.013
	*p*-value	.083		.241	.389	.004	.263	.013	.005	.373	.551	.899
Marital status	r	.469^a^	.122	1	.557^a^	.233^b^	.340^a^	.126	.097	.046	.070	-.202^b^
	*p*-value	.000	.241		.000	.023	.001	.222	.351	.659	.501	.050
Professional status	r	.473^a^	-.089	.557^a^	1	.419^a^	.512^a^	.304^a^	.262^b^	-.011	.202^b^	-.127
	*p*-value	.000	.389	.000		.000	.000	.003	.010	.917	.049	.221
Overall knowledge	r	.408^a^	-.289^a^	.233^b^	.419^a^	1	.593^a^	.631^a^	.684^a^	-.022	.075	-.051
	*p*-value	.000	.004	.023	.000		.000	.000	.000	.833	.468	.621
General knowledge	r	.335^a^	-.116	.340^a^	.512^a^	.593^a^	1	.396^a^	.414^a^	-.172	.003	-.023
	*p*-value	.001	.263	.001	.000	.000		.000	.000	.096	.978	.824
Trigger Knowledge	r	.232^b^	-.255^b^	.126	.304^a^	.631^a^	.396^a^	1	.915^a^	.022	.117	.108
	*p*-value	.023	.013	.222	.003	.000	.000		.000	.833	.261	.297
Prevention Knowledge	r	.187	-.286^a^	.097	.262^b^	.684^a^	.414^a^	.915^a^	1	.007	.134	.090
	*p*-value	.070	.005	.351	.010	.000	.000	.000		.944	.197	.387
Attitude	r	.105	-.092	.046	-.011	-.022	-.172	.022	.007	1	-.076	.212^b^
	*p*-value	.310	.373	.659	.917	.833	.096	.833	.944		.464	.039
Behavior	r	-.027	.062	.070	.202^b^	.075	.003	.117	.134	-.076	1	.171
	*p*-value	.792	.551	.501	.049	.468	.978	.261	.197	.464		.097
Refusal to treat	r	-.043	-.013	-.202^b^	-.127	-.051	-.023	.108	.090	.212^b^	.171	1
	*p*-value	.680	.899	.050	.221	.621	.824	.297	.387	.039	.097	

^a^ Correlation is significant at the 0.01 level (2-tailed)

^b^ Correlation is significant at the 0.05 level (2-tailed)

### Determinants of participants’ refusal to treat herpes labialis patients using binary regression

The determinants of refusal to treat patients with herpes labialis were marital status (*p* = 0.041, O.R = 0.113, 95 % C.I = 0.014–0.915) and attitude toward patients with herpes labialis (*p* = 0.018, O.R = 9.554, 95 % C.I = 1.463–62.380) (Table 4).

**Table 4:** Determinants of refusal to treat patients with herpes labialis among participants, using binary regression

Variable	B	S.E.	Wald	OR	95 % CI	*p*-value
Age	0.995	0.818	1.479	2.704	0.544−13.431	0.224
Sex	0.308	-0.745	0.172	1.361	0.316−5.857	0.679
Marital status	-2.179	1.067	4.174	0.113	0.014−0.915	0.041
Professional status	-1.135	0.933	1.478	0.321	0.052−2.003	0.224
Overall knowledge	-1.510	1.053	2.057	0.221	0.0281−.739	0.151
General knowledge	1.710	1.084	2.490	5.527	0.661−46.225	0.115
Trigger knowledge	1.929	2.578	0.560	6.882	0.04−1076.210	0.454
Prevention knowledge	-0.722	2.606	0.077	0.486	0.003−80.226	0.782
Attitude	2.257	0.957	5.558	9.554	1.463−62.380	0.018
Behavior	20.752	10430.160	0.000	1.029E9	0.000−.	0.998
Constant	-45.131	20860.321	0.000	0.000	-	0.998

Ref. age = ≤28 years; sex = male; marital status = unmarried; professional status = −undergraduate; overall knowledge = inadequate; general knowledge = inadequate; trigger knowledge = inadequate; prevention knowledge = inadequate; attitude = positive; behavior = inadequate

*OR* Odds Ratio, *CI* Confidence Interval, *B* intercept, *S.E.* standard error, *Wald* Wald chi-square test

## Discussion

Delivery of oral health care is the fundamental responsibility of dentists. However, these professionals are at risk for infections caused by various microorganisms such as *Mycobacterium tuberculosis*, HBV and HCV, staphylococci, streptococci, HSV type 1, HIV, mumps, influenza, and rubella [15, 16]. Herpes viruses shed in saliva can cause persistent infections in most exposed individuals, thus making such exposure a concern in dentistry [17]. Previous reports have indicated that the mechanisms, routes and risks of transmission of important viral pathogens encountered in dental practice are not clearly understood [18, 19]. In this study, more than half of the participants exhibited adequate herpes labialis trigger factor and transmission prevention knowledge, but only one-third (33.7 %) had adequate general knowledge about herpes labialis. These findings contrast with reports of good knowledge about the etiology, transmission pattern, and prevention of other bloodborne viral infections among most dentists in Italy and Pakistan [20, 21].

Preventing cross infection is considered an essential aspect of dental practice because disease transmission may occur this way in the dental health care setting. A prerequisite to understanding the need for infection control practices in dentistry is a sound knowledge of infectious diseases and their transmission potential in the oral health care setting [18]. In this study, adequate overall knowledge about herpes labialis was found in less than half (43.2 %) of participants. This is considered low for dental health care workers because inadequate knowledge may lead to suboptimal infection control practices with consequent infection in the form of herpetic whitlow and herpetic keratitis. This result also reflects insufficient understanding of herpes labialis among our participants. We found that less than 65 % of participants had good knowledge of HIV, HBV, and HCV, the bloodborne viral infections focused on by Kadeh et al. [22] in their study among Iranian dentists. The low proportion of adequate general, trigger factor, and transmission prevention knowledge of herpes labialis among our participants explains the low prevalence of adequate overall knowledge of herpes labialis. This is confirmed by the statistically significant positive correlation between overall knowledge, general, trigger factor, and transmission prevention knowledge in this study. The significant positive correlation between overall knowledge, age, sex, marital status, and professional status is consistent with reports of significant correlations between professional status and knowledge of HIV, HBV, and HCV infections among Italian and Iraqi dentists [20, 23].

The source of infection in dentistry may be the dental health care providers or patients with infectious diseases either in the prodromal stage or carrier (convalescent or asymptomatic) state. The reluctance of dentists to treat patients with infectious disease represents a major concern. Refusing treatment to patients whose infective status is definitive is not only unethical but also illogical because undiagnosed carriers of infectious disease pass undetected through dental health care settings daily [24, 25]. In this study, 16.8 % of participants reported a high likelihood of refusing treatment to patients with herpes labialis. Similarly, 16.0 % of Canadian and Indian dentists were unwilling to treat HIV-infected patients [4, 26]. However, Khosravanifard et al. [6, 7] reported 85.1 % and 44.4 % of Iranian dentists refused treatment for simulated HIV-positive patients and HBV-infected patients, respectively. This finding was higher than reported values among dental health care providers in Minnesota, United States (14.0 %) [27], Italian dentists (4.5–9.4 %) [5, 28], and dental hygienists (5.9 %) [29]. A total of 5.0 % of dental offices in Los Angeles County had an unlawful blanket policy of refusing dental services to people with HIV and AIDS [30]. Though not assessed in our study, this may be extended to other infectious diseases because medical students with career interests in surgical specialties (into which dentistry may be categorized) have indicated less willingness to provide care for patients with infectious diseases, presumably because of a perceived higher risk of exposure [31]. The successful resolution of concerns and enhancement of provider comfort levels with respect to treating patients with herpes labialis are therefore necessary to eliminate refusal to treat these patients and ensure appropriate patient care practices.

Although not statistically significant, young, unmarried, male undergraduate participants reported a higher likelihood of refusal to treat herpes labialis patients. There was a statistically significant positive correlation between attitudes and a tendency to refuse treatment to herpes labialis patients in this study. Refusal to treat patients with infectious disease results from a perceived stigma about treating such patients, discrimination, an absence of feeling ethical responsibility, a fear of being infected, and a lack of effective policies, proper knowledge, and appropriate facilities [4, 5, 26, 31, 32].

Using binary regression, the determinants of refusal to treat herpes labialis patients were marital status and attitude toward herpes labialis. This can be explained by the fact that non-professional attitudes, low optimism scores, and low comfort levels were among the best predictors of belief in the right to refuse treatment to HIV-infected patients [33]. Reducing concerns and enhancing providers’ comfort levels with respect to caring for patients with herpes labialis can help to reduce the tendency to refuse treatment to these patients. These were the parameters assessed in this study.

The findings of this study should be interpreted with caution because the analyzed information was self-reported and may be biased by underestimation, overestimation, and social desirability. In addition, the self-reported behavior of dental health care professionals may not necessarily reflect their actual behaviors [6, 7].

## Conclusion

 Data from this study revealed a high level of inadequate knowledge, negative attitudes, and reasonably adequate preventive behaviors regarding herpes labialis. One of every six dental health care workers studied reported having refused to treat patients with herpes labialis. Unmarried dental health care providers and those with negative attitudes toward patients with herpes labialis were more likely to refuse treatment to these patients.

## Additional files

Additional file 1:
**Questionnaire. **(DOCX 15 kb) Additional file 2:
**Database for the study.** (XLSX 19 kb)
