# Supplementary material for: Herpes labialis and Nigerian dental health care providers: knowledge, attitudes, behaviors, and refusal to treat
Source: BMC Health Serv Res. 2015 Sep 15;15:383. doi: 10.1186/s12913-015-1023-9 (PMC4572650; doi:10.1186/s12913-015-1023-9)
Supplement: Additional file 1: — Questionnaire. (DOCX 15 kb) [file 12913_2015_1023_MOESM1_ESM.docx]

**Additional file 1: Questionnaire**

**Herpes labialis and Nigerian dental healthcare providers: knowledge, attitude, behaviour and refusal to treat**

Please kindly respond to each statement with your most favourable answer. Thank you.

1. **Demographic characteristics**
2. Age (years): 20-22 [ ] 23-25 [ ] 26-28 [ ] 29-31 [ ] >31 [ ]
3. Gender: male [ ] female [ ]
4. Marital status: single [ ] married [ ]
5. Professional status: dental student [ ] House officer [ ] Resident doctor [ ]
6. **Knowledge of herpes labialis**

| Statement | Yes | No | I don’t know |
| --- | --- | --- | --- |
| 1. Herpes labialis are caused by virus |  |  |  |
| 1. Herpes labialis can be transmitted through tear |  |  |  |
| 1. Herpes labialis can be transmitted through saliva |  |  |  |
| 1. Herpes labialis can be transmitted through kissing |  |  |  |
| 1. Herpes labialis can be transmitted through sharing toothbrush |  |  |  |
| 1. Herpes labialis can be transmitted through sharing eating spoons and plates |  |  |  |
| 1. Herpes labialis can be transmitted through sharing make-up like lipsticks |  |  |  |
| 1. Herpes labialis can be transmitted through hand |  |  |  |
| 1. Herpes labialis can be transmitted through sexual intercourse |  |  |  |
| 1. Herpes labialis is usually a self-limiting disease. |  |  |  |
| 1. Treating patient with herpes labialis may cause herpetic whitlow in unprotected dentist |  |  |  |

1. **Which of the following is/are trigger (s) for herpes labialis?**

1. Fatigue Yes [ ] No [ ] 2. Fever Yes [ ] No [ ]

3. Strong sunlight Yes [ ] No [ ] 4. Menstruation Yes [ ] No [ ]

5. Stress Yes [ ] No [ ] 6. Hormone changes Yes [ ] No [ ]

7. Upper respiratory infection Yes [ ] No [ ] 8. Dental treatment Yes [ ] No [ ]

9. Extreme temperatures Yes [ ] No [ ] 10.Weakened immune system Yes [ ] No [ ]

1. **If a person has herpes labialis, should he or she do any of the following to avoid transmission to other people?**

1. Wash hands more frequently Yes [ ] No [ ] 2. Avoid shaking hands Yes [ ] No [ ]

3. Avoid kissing on lip/cheeks Yes [ ] No [ ] 4. Avoid sharing make-up like lipsticks Yes [ ] No [ ]

5. Avoid sexual intercourse Yes [ ] No [ ] 6. Avoid sharing eating spoons and plates Yes [ ] No [ ]

7. Tell partner that they have cold sore Yes [ ] No [ ]

When I treat a patient with cold sore:

| Statement | Strongly agree | agree | Neutral | disagree | Strongly disagree |
| --- | --- | --- | --- | --- | --- |
| 1. I feel comfortable |  |  |  |  |  |
| 1. I am concerned about getting it |  |  |  |  |  |
| 1. When I treat a patient with a cold sore |  |  |  |  |  |
| 1. I check the patient history |  |  |  |  |  |
| 1. I use proper infection control |  |  |  |  |  |
| 1. I want to educate them about cold sore |  |  |  |  |  |
| 1. I might not treat the patient on this day |  |  |  |  |  |
